# Association of Carotid Atherosclerosis With White Matter Hyperintensity in an Asymptomatic Japanese Population: A Cross-Sectional Study

**DOI:** 10.3389/fcvm.2021.665573

**Published:** 2021-04-29

**Authors:** Li Zhang, Quan Zhou, Li Hua Shao, Jun Wen, Jun Xia

**Affiliations:** ^1^Department of Neurology, The First People's Hospital of Changde, Changde, China; ^2^Department of Science and Education, The First People's Hospital of Changde, Changde, China; ^3^Department of Neurosurgery, The First People's Hospital of Changde, Changde, China

**Keywords:** carotid artery plaque score, carotid atherosclerosis, carotid plaque number, white matter hyperintensity, cross-sectional study

## Abstract

**Objective:** A limited number of scholars concentrated on the relationship between carotid atherosclerosis (CAS) and white matter hyperintensity (WMH) (i.e., CAS-WMH relationship). The current research aimed to clarify the CAS-WMH relationship in Japanese population.

**Methods:** All participants underwent MRI of head and ultrasonography of the carotid artery. WMH was diagnosed from MRI results. The carotid ultrasound findings, carotid artery plaque score (PS), and plaque number (PN) could be achieved to indicate the severity of CAS. We also employed multivariate logistic regression models to estimate the CAS-WMH relationship. Interaction and stratified analyses were undertaken on the basis of a number of factors (e.g., gender, age, smoking status, drinking habit, and history of chronic diseases).

**Results:** A total of 1,904 Japanese subjects were included, and the prevalence of WMH was 54.8% (1,044/1,904). It was unveiled that frequency of CAS was greater in cases with WMH. In a fully adjusted model, high PS was associated with the frequency of WMH, followed by high PN. Further analyses revealed a dose-response relationship between PS and incidence of WMH.

**Conclusion:** PS and PN exhibited the greatest influences on determining the frequency of WMH, highlighting the potentially important pathophysiological role of large artery atherosclerosis in intensifying WMH.

## Introduction

Cerebral white matter hyperintensity (WMH) is an extremely frequent finding on magnetic resonance imaging (MRI) or computed tomography (CT) of brain in cases with stroke and dementia (1). Although a number of scholars attempted to concentrate on WMH, efforts were made to identify association between WMH and factors that may influence WMH (2, 3). Previously, pathologic thickening or necrosis of the vessel wall were noted to affect WMH (4, 5), while no information could be attained justifying how large-artery atherosclerosis may influence WMH.

Recently, increasing evidence has indicated a close association between carotid atherosclerosis (CAS) and WMH (i.e., CAS-WMH relationship) (6, 7). Shu et al. (8) demonstrated that CAS could lead to WMH by inducing cerebral hypoperfusion. Insufficient cerebral perfusion due to CAS may affect the severity of WMH and increase the frequency of WMH (9). Moreover, WMH may be partially reversible in patients with carotid artery stenosis (10). These findings highlight an important role of CAS in WMH as an intermediate factor, although this conclusion remains controversial.

Carotid ultrasonography, a non-invasive examination, can be utilized to identify CAS in the early stages. Numerous researches have used intima-media thickness (IMT) as a surrogate marker of carotid stiffness, while few reports have concentrated on plaque score (PS) and plaque number (PN). PS may more precisely represent the atherosclerotic condition of the carotid artery than IMT because a wider range of observations are required to obtain PS compared to IMT, which suggests the superiority of PS in indicating CAS (11).

In the present study, PS and PN were assessed as indicators of CAS to investigate the associations between the severity of CAS and the incidence of WMH in Japanese population. It also was attempted to indicate how vascular risk factors may influence the mentioned association with the assistance of stratified analysis.

## Methods

### Collection of Data

The DATADRYAD database was employed to attain data. We also made an effort to cite the Dryad data package following protocols released in the Dryad Digital Repository (Tokyo, Japan).

### Study Design and Participants

Our research was undertaken via Shinkawa et al.'s findings (12). Their cross-sectional study involved a comprehensive medical checkup of 1,904 asymptomatic participants, including 988 men and 916 women, who were admitted to Shin Takeo Hospital between April 1, 2016, and October 31, 2017, some patients underwent head MRI, blood tests and carotid ultrasonography, and completed standard questionnaires all within this period. Exclusion of subjects was undertaken on the basis of criteria outlined below: presence of (1) a number of devices frequently utilized surgically; (2) a psychiatry-dependent disease; (3) paralysis or a history of stroke, traumatic brain injuries, and other definitive encephalopathies; and (4) poorly recorded carotid ultrasonograms precluding measurement; (5) subjects who were unintended to cooperate. Procedures related to consent to participate and confirming the protocol were detailed in the native manuscript (12).

### Extraction and Processing of Data

The comprehensive medical examination included a general inspection (age, sex, body mass index, diastolic blood pressure [DBP], systolic blood pressure [SBP], presence of visceral steatosis [for the determination of metabolic syndrome]), blood and biochemical tests, and a questionnaire on medical history (antihypertensive medicine, antidiabetic medicine, and cholesterol-lowering medications), and lifestyle characteristics, including drinking habits, drinking volume, and smoking habits. Fasting blood samples were analyzed for low-density lipoprotein cholesterol (LDL-C), high-density lipoprotein cholesterol (HDL-C), ratio of LDL-C to HDL-C, triglyceride, fasting plasma glucose, and glycated hemoglobin (HbA1c).

As described previously (12), it was attempted to extract data outlined below: smoking habits (smoking >100 cigarettes or subsequent smoking for 6 months as well as attempting to smoke in the recent 1 month; drinking status (rarely, sometimes, and daily); and drinking volume (with respect to quantify the consumption of alcoholic beverage/day) in form of <180, 180–360, 360–540 or >540 mL. Hypertension was defined as SBP >140 or DBP > 90 mmHg (or both) on at least two diverse conditions. We also attempted to define hyperlipidemia on the basis of diverse criteria (total plasma cholesterol level >5.7 mmol/L, or levels of LDL-C, HDL-C, and triglyceride >3.4 mmol/L, lower than 1.0 mmol/L, and higher than 1.7 mmol/L, respectively. Diabetes mellitus was defined as a history of diabetes, current anti-diabetic treatment, or if fasting plasma glucose ≥6.1 mmol/L or random blood glucose of ≥11.1 mmol/L.

The carotid arteries were imaged using the LOGIQ S7 Expert and Aplio 400 on the basis of released protocols. We also attempted to carefully search for all carotid artery-dependent associations [i.e., central side, peripheral side, bifurcation of the common carotid artery (CCA) and central site of the internal carotid artery]. The peak value of IMT equal or higher than 1.1 mm was employed for definition of plaque. It was also made an effort to sum thicknesses of plaques for calculation of PS. In the head MRI, MRI scanners were utilized. Estimation of the frequency of WMH could be undertaken with the aid of images of T1- and T2-weighted, as well as fluid-attenuated inversion recovery (FLAIR). WMH was defined as clear hyperintensity in the white matter region, relative to the surrounding white matter on FLAIR images. Two independent experienced scholars analyzed MRI findings in a blinded fashion.

### Statistical Analysis

Expression of continuous variables was in form of mean ± SD (for normally distributed outcomes) or median (IQR) (for normally distributed outcomes), and presentation of categorical variables was undertaken as frequencies and percentages. First, one-way analysis of variance and the Chi-square tests we employed to compare continuous variables and categorical variables, respectively. The association of PS or PN with WMH was assessed using multivariable logistic regression models. Non-adjusted, minimally adjusted, and multivariate-adjusted models were employed. The covariances were adjusted according to the following principle: (1) in Model II covariates whose initial regression coefficients changed by at least 10% were included; (2) in Model III covariates in Model II plus previously reported classical vascular risk factors were included; and (3) since the prevalence of WMH strongly increases with age and varies with sex, these two covariates were included in all adjusted models. In another separate analysis, PN was included as a continuous variable. Unadjusted and adjusted odds ratios (ORs) with 95% confidence intervals (CIs) were calculated. We also used a generalized additive model to identify the non-linear relationships between PS and the frequency of WMH. Interaction and stratified analyses were conducted according to age group (three equal groups), sex, smoking status, drinking status, and history of chronic disease as previously described. We employed R programming and Empower Stats software to conduct the analyses. We set the significance level to *P* < 0.05.

## Results

### Subjects' Features

**Figure 1:**
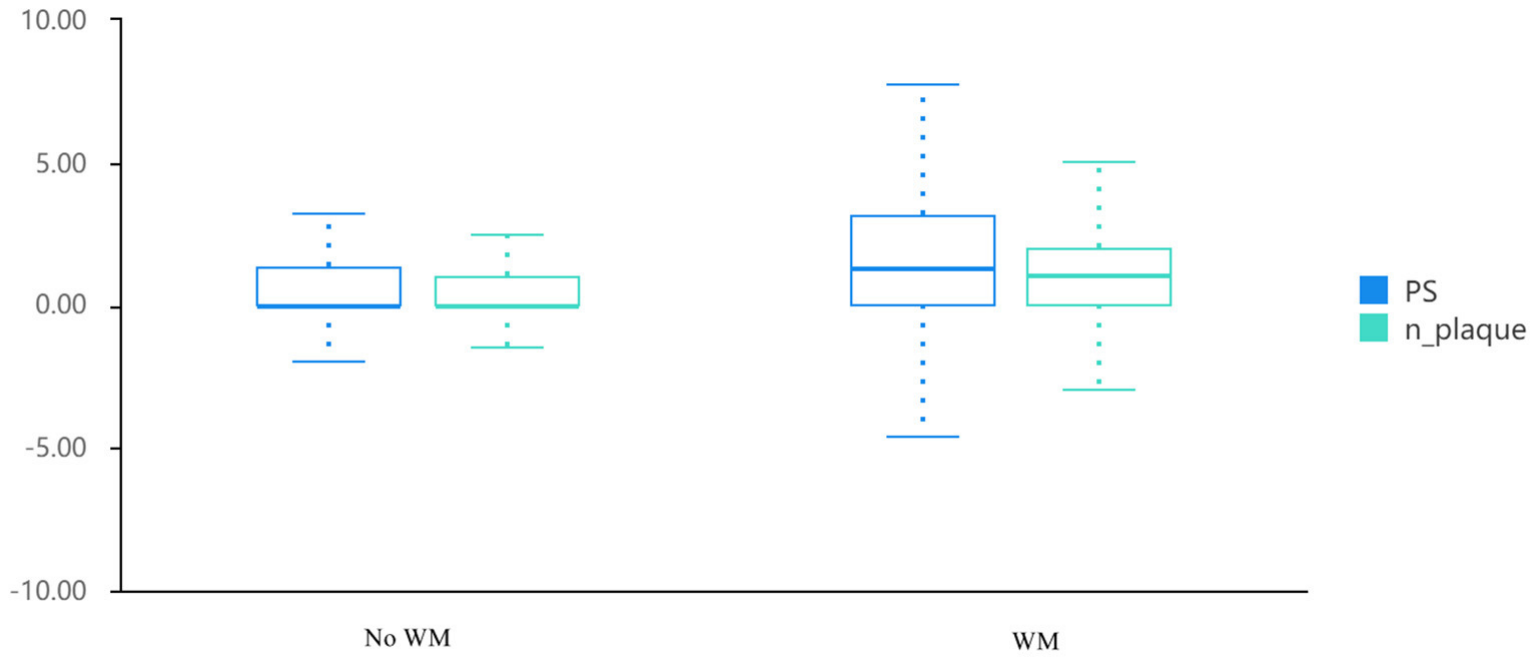
Distribution of PS and PN in patients with white matter hyperintensity and controls. PS, plaque score; PN, plaque number.

Among the 1,904 participants with a mean age of 56.4 ± 11.5 years old, 1,044 (54.8%) were diagnosed with cerebral WMH from their MRI results. Table 1 illustrates the comparisons' outcomes between WMH and non-WMH groups. Several factors were significantly different between the two groups. The WMH group showed higher age, lower male proportion, smoking habit, and drinking habit ratio, higher incidences of hypertension, hyperlipidemia, and diabetes mellitus, and a higher metabolic syndrome ratio, HDL-C, fasting plasma glucose, HbA1c, SBP and DBP compared to the non-WMH group. No significant difference was detected in levels of triglyceride and LDL-C between the above-mentioned groups. The main findings of carotid ultrasound were markedly greater in the WMH group than those in the non-WMH group [1.3 (0.0, 3.1) and 1.0 (0.0, 2.0), and 0.0 (0.0, 1.3) and 0.0 (0.0, 1.0), respectively; Table 1 and Figure 1].

**Table 1:** Comparison of clinical parameters between WML and non-WMH patients.

**Variables**	**All participants**	**Non-WMH**	**WMH**	***p***
**General**				
Participants (*n*)	1,904	860	1,044	
Age (years)	56.4 ± 11.5	50.0 ± 10.8	61.7 ± 9.2	<0.001
male, n (%)	988 (51.9)	497 (57.8)	491 (47)	<0.001
BMI (Kg/m^2^)	23.2 ± 3.4	23.1 ± 3.4	23.2 ± 3.4	0.774
HT, *n* (%)	462 (24.3)	109 (12.7)	353 (33.8)	<0.001
DM, *n* (%)	139 (7.3)	32 (3.7)	107 (10.2)	<0.001
DL, *n* (%)	315 (16.5)	82 (9.5)	233 (22.3)	<0.001
Smoking habit, *n* (%)	336 (17.6)	197 (22.9)	139 (13.3)	<0.001
Drinking habit, *n* (%)				0.029
rarely drink	796 (41.8)	331 (38.5)	465 (44.5)	
sometimes	565 (29.7)	269 (31.3)	296 (28.4)	
everyday	543 (28.5)	260 (30.2)	283 (27.1)	
Amount of drinking per day, *n* (%)				<0.001
Less than 180 mL	1224 (64.3)	504 (58.6)	720 (69)	
180–360 mL	467 (24.5)	237 (27.6)	230 (22)	
360–540 mL	158 (8.3)	89 (10.3)	69 (6.6)	
More than 540 mL	55 (2.9)	30 (3.5)	25 (2.4)	
Metabolic syndrome, *n* (%)				<0.001
No	1429 (75.1)	687 (79.9)	742 (71.1)	
Reserve	196 (10.3)	79 (9.2)	117 (11.2)	
Yes	279 (14.7)	94 (10.9)	185 (17.7)	
**Biochemical data**				
LDL (mg/dl)	120.9 ± 30.4	119.6 ± 31.6	121.9 ± 29.4	0.109
HDL (mg/dl)	61.1 ± 15.4	59.5 ± 14.7	62.5 ± 15.8	<0.001
LH (%)	2.1 ± 0.8	2.1 ± 0.8	2.1 ± 0.7	0.064
TG (mg/dl)	89.0 (64.0, 129.0)	87.0 (62.5, 129.0)	91.0 (66.8, 129.0)	0.299
HbA1c (%)	5.7 (5.4, 5.9)	5.6 (5.4, 5.8)	5.7 (5.5, 6.0)	<0.001
BS (mg/dl)	101.0 (95.0, 107.0)	99.5 (95.0, 105.0)	102.0 (95.0, 110.0)	<0.001
SBP (mmHg)	123.9 ± 18.4	120.0 ± 16.6	127.1 ± 19.3	<0.001
DBP (mmHg)	73.9 ± 12.2	72.4 ± 11.6	75.0 ± 12.5	<0.001
**Carotid data**				
PN, *n* (%)				<0.001
0	1241 (65.2)	666 (77.4)	575 (55.1)	
1	337 (17.7)	120 (14)	217 (20.8)	
2	203 (10.7)	54 (6.3)	149 (14.3)	
3	79 (4.1)	15 (1.7)	64 (6.1)	
4	33 (1.7)	5 (0.6)	28 (2.7)	
5	6 (0.3)	0 (0)	6 (0.6)	
6	4 (0.2)	0 (0)	4 (0.4)	
8	1 (0.1)	0 (0)	1 (0.1)	
PS	0.0 (0.0, 1.6)	0.0 (0.0, 1.3)	1.3 (0.0, 3.1)	<0.001

*Values are given as mean ± standard deviation, medians with interquartile range or number (%). HT, hypertension; DM, diabetes mellitus; DL, dyslipidemia; BMI, ankle-brachial index; PS, carotid plaque score; PN, plaque number; LDL-C, low-density lipoprotein cholesterol; HDL-C, high-density lipoprotein cholesterol; LH, quotient of LDL-C and HDL-C; TG, Triglyceride; HbA1c, hemoglobin A1c; BS, blood glucose level; SBP, systolic blood pressure; DBP, diastole blood pressure*.

### Association of PS and PN With WMH

We attempted to conduct multivariable logistic regression analysis for indicating factors that might contribute to the frequency of WMH (Table 2). In the non-adjusted model, odds ratio (OR) was 1.36, and the range of 95% confidence interval (CI) was 1.28 to 1.45. It could be interpreted that a unit increase of PS exhibited a correlation with a 36% increase of incidence of WMH. It was noted that every one unit increase in PS could be associated with a 25% elevation in frequency of WMH. Besides, high PN was also identified to contribute to the incidence of WMH [OR, 1.36 (95% CI: 1.28–1.54)]. To ensure the robustness of our results, we could convert PS value into a categorical variable according to the cut-off points (0, 2.5), and we calculated the *P*-value for trend. There was an increased risk for the prevalence of WMH as the cutoff point for PS was elevated (*P* (for trend) <0.001). Participants who had a great PS experienced an elevated risk of the incidence of WMH [OR, 3.68 (95% CI: 2.79–4.85)].

**Table 2:** Association between PS and incidence of White matter hyperintensity in multiple regression model.

**Variable**	**Non-adjusted Model**		**Model I**		**Model II**		**Model III**	
	**OR (95% CI)**	***P*-value**	**OR (95% CI)**	***P*-value**	**OR (95% CI)**	***P*-value**	**OR (95% CI)**	***P*-value**
PS	1.36 (1.28, 1.45)	<0.001	1.11 (1.04, 1.19)	0.002	1.1 (1.02, 1.17)	0.009	1.1 (1.03, 1.18)	0.006
**PS Tertiles**								
T1(=0)	1		1		1		1	
T2(≥0.1, <2.5)	2.20 (1.71, 2.82)	<0.001	1.19 (0.89, 1.59)	0.233	1.12 (0.84, 1.51)	0.430	1.13 (0.85, 1.52)	0.398
T3(≥2.5)	3.68 (2.79, 4.85)	<0.001	1.55 (1.12, 2.14)	0.008	1.44 (1.03, 2.00)	0.031	1.48 (1.04, 2.07)	0.023
P for trend		<0.001		0.007		0.033		0.025
PN	1.78 (1.59, 2.00)	<0.001	1.24 (1.09, 1.40)	<0.001	1.20 (1.06, 1.36)	0.004	1.21 (1.07, 1.38)	0.003

*PS, carotid plaque score. PN, plaque number, SBP, systolic blood pressure, DBP, diastole blood pressure.CI, confidence interval. OR, odds ratio*.

*Model III adjusted for Age, Sex, BMI, SBP, DBP, HT, DM, DL, HDL, BS, HAB1C, Smoking habit and Drinking habit*.

### Analysis of a Dose-Response Relationship

**Figure 2:**
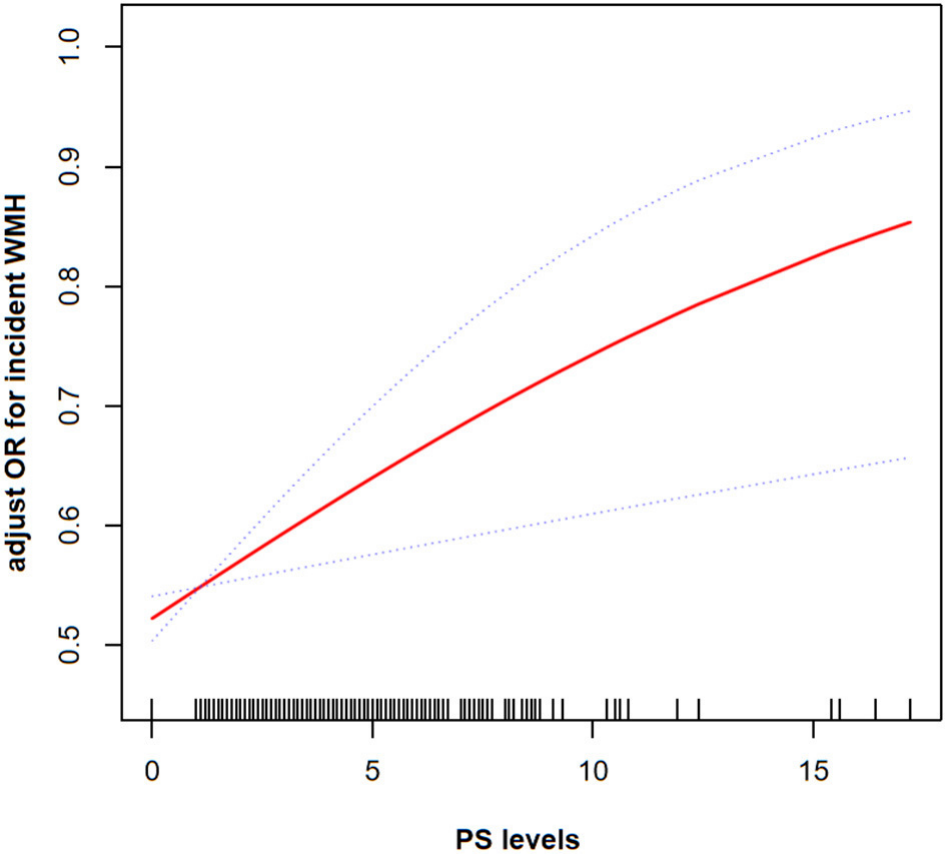
The smoothing curves illustrating the relationship between PS and frequency of WMH. A dose-response relationship between them was detected after adjusting for gender, age, SBP, DBP, BMI, HT, DL, DM, HDL, BS, HAB1C, smoking history, and drinking status.

The generalized additive model (GAM) model was used to indicate the existence of a non-linear relationship between PS and incidence of WMH. After adjusting for vascular risk factors, a non-significant, moderate, beneficial effect on correlation of high PS with incidence of WMH was identified (Figure 2).

### Subgroup Analysis

**Figure 3:**
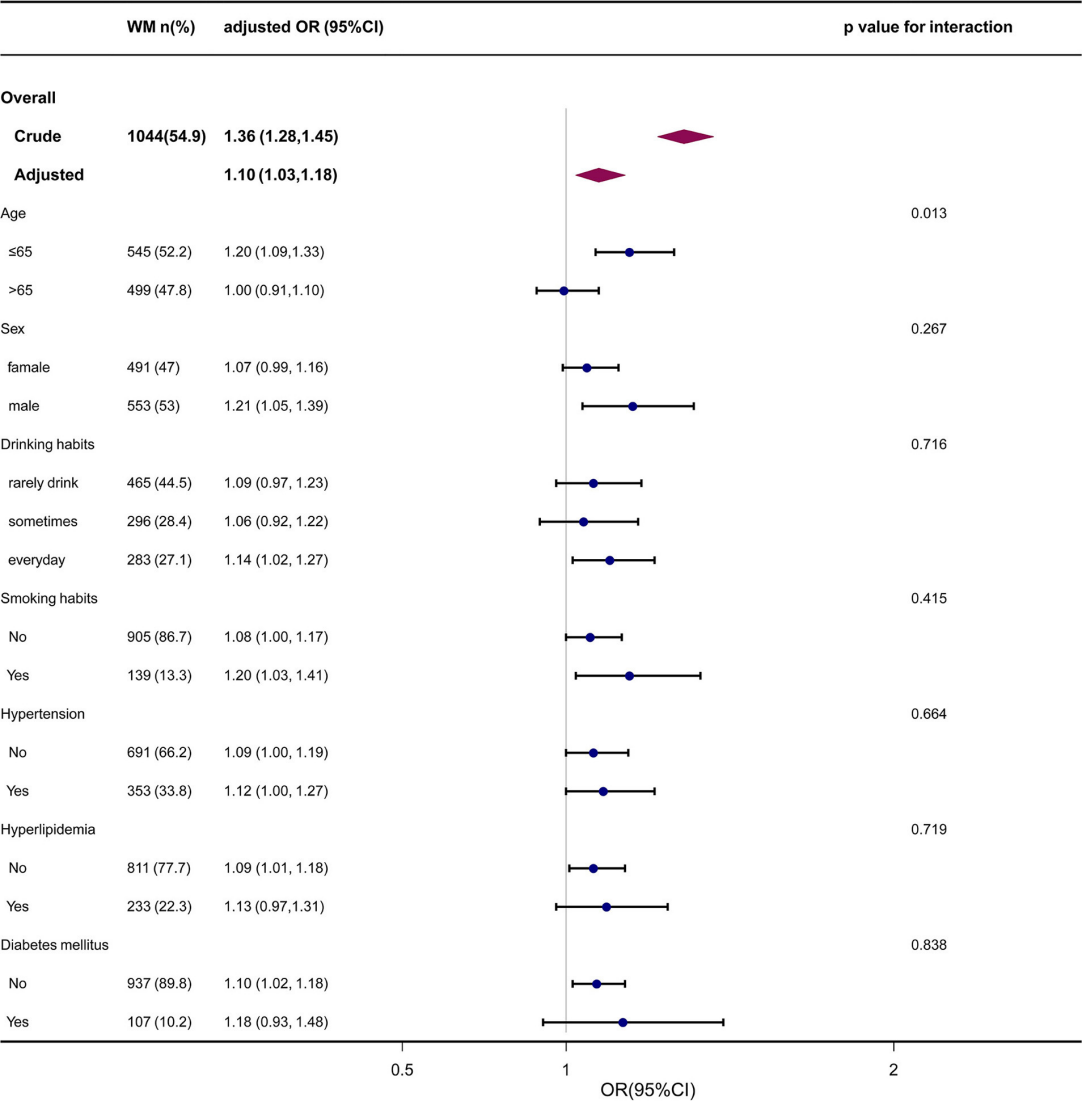
The forest plot of the association between PS and WMH according to baseline characteristics. *P*-value represents the likelihood of interaction between the variable and the PS. PS, carotid plaque score, WMH, white matter hyperintensity; OR, odds ratio; CI, confidence interval.

Age, gender, smoking status, drinking habit, and history of chronic diseases were identified as confounders of the PS-WMH association. To determine whether the association between PS and frequency of WMH could be detected in diverse subgroups, we made an effort to carry out further analyses. Subgroup analyses stratified by gender, age, smoking status, drinking habit, and history of chronic diseases yielded consistent outcomes (Table 3 and Figure 3). The interaction analysis revealed that age could make a link between PS and incidence of WMH. The incidence of WMH was higher in those cases who aged <65 years old, while we did not detect a significant association in cases who aged > 65 years old. No noticeable interaction was identified for sex, drinking habit, smoking status, history of hypertension, hyperlipidemia, and diabetes mellitus.

**Table 3:** Association between PS and White matter hyperintensity according to baseline characteristics.

**Subgroup**	**No WM**	**WM**	**OR (95%CI)**		***p*-value for interaction**
	***n* (%)**	***n* (%)**	**No adjusted**	**Mutually adjusted^a^**	
Age					0.013
<65	762 (88.6)	545 (52.2)	1.40 (1.28, 1.53)	1.20 (1.09, 1.33)	
≥65	98 (11.4)	499 (47.8)	1.0 (0.92, 1.09)	1.00 (0.91, 1.10)	
Sex					0.267
Female	497 (57.8)	491 (47.0)	1.38 (1.28, 1.49)	1.07 (0.99, 1.16)	
Male	363 (42.2)	553 (53.0)	1.53 (1.33, 1.75)	1.21 (1.05, 1.39)	
Drinking habits					0.716
Rarely drink	331 (38.5)	465 (44.5)	1.32 (1.19, 1.48)	1.09 (0.97, 1.23)	
Sometimes	269 (31.3)	296 (28.4)	1.39 (1.23, 1.57)	1.06 (0.92, 1.22)	
Everyday	260 (30.2)	283 (27.1)	1.41 (1.27, 1.57)	1.14 (1.02, 1.27)	
Smoking habits					0.415
No	663 (77.1)	905 (86.7)	1.34 (1.25, 1.44)	1.08 (1.00, 1.17)	
Yes	197 (22.9)	139 (13.3)	1.48 (1.29, 1.71)	1.20 (1.03, 1.41)	
HT					0.664
No	751 (87.3)	691 (66.2)	1.36 (1.26, 1.48)	1.09 (1.00, 1.19)	
Yes	109 (12.7)	353 (33.8)	1.15 (1.04, 1.28)	1.12 (1.00, 1.27)	
DL					0.719
No	778 (90.5)	811 (77.7)	1.36 (1.27, 1.47)	1.09 (1.01, 1.18)	
Yes	82 (9.5)	233 (22.3)	1.19 (1.04, 1.36)	1.13 (0.97, 1.31)	
DM					0.838
No	828 (96.3)	937 (89.8)	1.34 (1.26, 1.44)	1.10 (1.02, 1.18)	
Yes	32 (3.7)	107 (10.2)	1.23 (1.01, 1.51)	1.18 (0.93, 1.48)	

a *Each stratification adjusted for all the factors (Age, Sex, SBP, DBP, BMI, HT, DM, DL, HDL, BS, HBA1C, Smoking habit and Drinking habit) except the stratification factor itself*.

## Discussion

The current cross-sectional study aimed to clarify the CAS-WMH relationship in Japanese population. The findings of the current research indicated that functional markers of carotid atherosclerosis (high values of PN and PS on the carotid ultrasonography) noticeably influenced determining the frequency of WMH, independent of demographic and vascular risk factors. We additionally revealed a dose-response relationship between PS and incidence of WMH. Even after adjusting for vascular risk factors, subgroup analyses stratified by multiple variables yielded consistent results. The CAS-WMH relationship demonstrated that structural carotid artery remodeling may be important in the pathophysiology of WMH, and it is consistent with that reported previously. Numerous researches have also described a positive association between the number or characteristics of the carotid plaque and incidence of WMH (6, 7, 13–15). The Rotterdam study recruited healthy adults who aged 65–85 years old and investigated an association between WMH and non-invasively assessed atherosclerosis, and found that CAS could be correlated to WMH (6). de Leeuw et al. reported a graded relationship between IMT severity and the presence of plaques in the carotid artery with periventricular WMH, rather than with subcortical WMH (7). Fernando et al. examined a relationship between carotid plaques and severe WMH at the 4-year follow-up, and showed that indicators of CAS, IMT and plaques, were associated with the frequency of severe WMH (14). Moreover, a recent meta-analysis revealed that WMH tended to be associated with carotid plaques and plaques or IMT increased with elevation of WMH severity (13).

Previous reports clearly showed that the high IMT observed by carotid ultrasonography is strongly associated with WMH incidence. However, the validity of atherosclerosis measurements should be first clarified. Increased carotid IMT according to the Mannheim consensus may not be an indicator of atherosclerosis (16). Increased carotid IMT without plaques, as determined by ultrasonography, is only associated with cardiovascular risk factors (17). Moreover, neither progression (18) nor regression (19) of IMT predicted the influences of cardiovascular risk factors. In a 6-year follow up study, IMT did not predict coronary risk, whereas total plaque area was noted as a robust predictor of coronary risk (20). In contrast, PS was associated with coronary heart disease (21), and PS more closely represented the atherosclerotic condition of the carotid artery compared to IMT (11) because of its wider range of observations in terms of carotid arteries. In the current research, it was unveiled how high PS could be markedly associated with WMH, and we also identified a dose-response relationship between frequency of WMH and PS, independent of factors mentioned earlier. It is noteworthy that we uniquely attempted to investigate the dose-response relationship that may further confirm WMH-CAS relationship.

The mechanisms underlying the WMH-CAS association are speculative. Factors that may interfere with cerebral blood flow, such as unstable microatheroma at the origin of perforator arteries, may play a role (22). Cerebral emboli originating from ruptured or unstable carotid plaques might intensify development of WMH, although they may not be major contributors to a population-based setting (1, 22). Other mechanisms could include large-artery hypertrophic remodeling and capillary rarefaction, continually and passively, to highly pulsatile pressure and flow, causing chronic ischemic lesions (23, 24). Non-stenotic CAS, defined as IMT or carotid plaque, was associated with larger ipsilateral intracranial artery remodeling (25), which in turn may distally influence hemodynamics, thereby affecting intracranial arterial remodeling. There may be a possible balance between changes in the cardiovascular homeostasis of the brain tissue, which may lead to white matter lesions when lost (26). Otherwise, the carotid plaque may also reflect the overall effect of the shared vascular risk factors, or lifetime exposure to factors, including age, smoking status, and atrial fibrillation, which are also associated with WMH according to previous researches (27, 28). A possible explanation is that atrial fibrillation could modulate pathogenesis of WMH through chronic silent microembolization (27, 29).

The outcomes of the current research unveiled how PS could be associated with the frequency of WMH, while age factor was excluded. It was illuminated that a high PS was positively associated with WMH incidence in subjects aged <65 years old, rather than in senior adults. As the most significant risk factor, a number of scholars concentrated on the role of aging in WMH was documented noteworthy (30), and older subjects typically experience a higher incidence of WMH; thus, some “non-ischemic” WMH may partly explain the results. Additionally, “non-ischemic” WMH may simply be a CT or MRI manifestation of the dilation of the perivascular spaces, which were filled with cerebrospinal fluid or atrophic changes that include gliosis or the loss of the myelinated axons seen in the normal, aging brain (31, 32). Consequently, bias was inevitable when we analyzed the association between PS and the prevalence of WMH in senior subjects.

The strengths of our study include the use of a large cohort of asymptomatic individuals in Japan in a single-center, which realized collecting data systematically and uniformly. In addition, strict statistical adjustments were employed to minimize unmeasured confounding. Moreover, standardized ultrasound scanning and PS were utilized as reliable measures of atherosclerosis rather than IMT used in a previous research (11). Finally, experienced scholars analyzed MRI findings in a blinded fashion, which might attenuate information bias.

The shortcomings of the current research should be acknowledged. First, no data regarding the severity of WMH could be presented. Second, this was a cross-sectional study and no data on correlation of the aggravation of CAS and the extension of WMH could be provided. Third, blood tests and ultrasonic scanning were undertaken on the basis of a single measurement, which might cause regression dilution bias. In addition, differentiating the “non-ischemic” or “natural” hyperintensity from the WMH region of interest is typically difficult, and a software could better indicate the spectrum of small vessel illnesses. Finally, as the current research was undertaken in a single-center, generalizability of the results is questionable. Hence, it is essential to conduct further researches to eliminate the above-mentioned shortcomings.

## Conclusions

The current research enabled us to identify how an increase in values of PS and PN could be independently correlated with a higher incidence of WMH in an asymptomatic population. It was confirmed that PN and PS exhibited the greatest influences on determining the incidence of WMH, independent of demographic and vascular risk factors. Furthermore, a dose-response relationship between PS and incidence of WMH was noted. Our findings highlighted the potentially important pathophysiological role of large artery atherosclerosis in intensifying WMH. However, the mentioned outcomes should be confirmed by conducting further researches.

## Data Availability Statement

The datasets presented in this study can be found in online repositories. The names of the repository/repositories and accession number(s) can be found in the article/Supplementary Material.

## Ethics Statement

The studies involving human participants were reviewed and approved by the ethical review committee of Shin Takeo Hospital. The patients/participants provided their written informed consent to participate in this study.

## Author Contributions

LZ contributed to the drafting of the manuscript and analysis and interpretation of the data. JX contributed to the conception, critical revision of the manuscript, analysis, and interpretation of data, and approved the final version of the submitted manuscript. LZ and JX read and approved the final manuscript. All authors contributed to the article and approved the submitted version.

## Conflict of Interest

The authors declare that the research was conducted in the absence of any commercial or financial relationships that could be construed as a potential conflict of interest.

## Abbreviations

CT: computed tomography
DBP: diastolic blood pressure
FLAIR: fluid-attenuated inversion recovery
HbA1c: hemoglobin A1c
HDL-C: high-density lipoprotein cholesterol
IMT: intima-media thickness
LDL-C: low-density lipoprotein cholesterol
MRI: magnetic resonance imaging
PN: plaque number
PS: carotid plaque score
SBP: systolic blood pressure
WMH: white matter hyperintensity.
