# Supplementary material for: Association of Carotid Atherosclerosis With White Matter Hyperintensity in an Asymptomatic Japanese Population: A Cross-Sectional Study
Source: Front Cardiovasc Med. 2021 Apr 29;8:665573. doi: 10.3389/fcvm.2021.665573 (PMC8118638; doi:10.3389/fcvm.2021.665573)
Supplement: Supplementary file 2 [file Table_1.docx]

| Supplemental table 1. Association between the symptomatic carotid atherosclerosis and incidence of White matter hyperintensity in multiple regression model | | | | | | | | |
| --- | --- | --- | --- | --- | --- | --- | --- | --- |
| Variable | Non-adjusted Model | | Model I | | Model II | | Model III | |
|  | OR (95% CI) | P-value | OR (95% CI) | P-value | OR (95% CI) | P-value | OR (95% CI) | P-value |
| Non-CP | 1 |  | 1 |  | 1 |  | 1 |  |
| CP | 2.80(2.29, 3.42) | <0.001 | 1.33 (1.05, 1.70) | 0.020 | 1.25 (0.98, 1.60) | 0.078 | 1.25 (0.98, 1.60) | 0.075 |
| Abbreviations: CP, carotid plaque.. SBP, systolic blood pressure, DBP, diastole blood pressure.CI, confidence interval. OR, odds ratio.  Model Ⅰadjusted for Age and Sex.  Model Ⅱadjusted for Age, Sex, SBP, DBP, HT, DM, DL and Drinking habit.  Model III adjusted for Age, Sex, BMI, SBP,DBP,HT, DM,DL，Smoking habit and Drinking habit. | | | | | | | | |
|  |  |  |  |  |  |  |  |  |
|  |  |  |  |  |  |  |  |  |
|  |  |  |  |  |  |  |  |  |
|  |  |  |  |  |  |  |  |  |
|  |  |  |  |  |  |  |  |  |
